# Iatrogenic Cerebrospinal Fluid Leak in Endoscopic Sinus Surgery: Topographical Map and Influence of Skull Base Asymmetry

**DOI:** 10.3390/jpm14030226

**Published:** 2024-02-21

**Authors:** Alessandro Vinciguerra, Isabelle Dohin, Antonio Daloiso, Francesco Boaria, Morgane Marc, Benjamin Verillaud, Florian Chatelet, Philippe Herman

**Affiliations:** 1 Otorhinolaryngology and Skull Base Center, AP-HP, Hospital Lariboisière, 75010 Paris, France; francesco.boaria@gmail.com (F.B.); morgane.marc@aphp.fr (M.M.); chatelet.florian@gmail.com (F.C.); philippe.herman099@gmail.com (P.H.); 2 Unit of Otorhinolaryngology-Head and Neck Surgery, ASST Spedali Civili Brescia, Department of Medical and Surgical Specialties, Radiological Sciences and Public Health, University of Brescia, 25121 Brescia, Italy; isabelle.dohin@hotmail.com; 3 Unit of Otorhinolaryngology-Head and Neck Surgery, Department of Neurosciences, University of Padua, 35122 Padua, Italy; antoniodaloiso96@gmail.com

**Keywords:** cerebrospinal fluid leak, iatrogenic, skull base, intracranial hypertension

## Abstract

Background: Iatrogenic cerebrospinal fluid leak (iCSF-L) is a major complication of endonasal surgeries whose occurrence is always a potential adverse event due to anatomical variation/asymmetry of the skull base (SB). The aim of this manuscript is to provide a topographical map of iCSF-L and to investigate the role of SB asymmetry in iCSF-L occurrence. Methods: In this retrospective study, the location of iCSF-L dural defect was studied and compared to patients affected by spontaneous and post-traumatic CSF-L. Considering only iCSF-L, after having collected the SB asymmetry data, the Keros, Gera, distance of the anterior ethmoidal artery from the SB, frontal sinus pneumatization, and Thailand–Malaysia–Singapore score classifications were compared to a control group of patients. Results: A total of 153 CSF-Ls (103 spontaneous, 37 iatrogenic, and 13 traumatic) were included. A significant association was noted (*p* < 0.001) between the nature of the CSF-L and the areas involved. Considering iCSF-Ls, only the Gera classification was significantly different (*p* < 0.05) and the most reliable in predicting the risk of dural transgression (AUC = 0.719). Conclusions: ICSF-Ls present peculiar regional SB involvement with the cribriform plate, with the ethmoidal roof being the most involved. After having assessed the asymmetry of the SB, the Gera classification was the most reliable one.

## 1. Introduction

Iatrogenic cerebrospinal fluid leak (iCSF-L) is a major complication of endoscopic endonasal surgeries occurring in 0.5–3% of procedures [1]. In general, 50% of these cases present intra-operative or immediate post-operative symptoms of iCSF-L, whereas in the other 50%, the occurrence is delayed from 1 week to 1 month, mainly due to wound contraction, flap devascularization, or necrosis [1,2]. This complication poses significant risks to the patient’s post-operative recovery, and revision surgery is often required, increasing both costs and potential malpractice litigation [3,4]. 

As a general rule, extensive anatomical knowledge of the sinonasal compartment and increased surgical expertise help prevent iCSF-L, although it always represents a potential adverse event mainly due to anatomical variations in the skull base [5,6]. Indeed, in the literature, several authors have proposed different classifications to stratify the risk of iCSF-L based on anatomical variations in the principal structures known to be at major risk of dural transgression, namely the lateral lamella and ethmoidal roof [5,7,8,9,10], which are the thinnest and most vulnerable structures of the anterior skull base and, as a result, the most common sites of inadvertent skull base violation [6,11]. Moreover, in recent reports, the role of skull base asymmetry has been investigated, underlying its potential clinical relevance in iCSF-L [12]. In fact, even if this feature has been described in the abovementioned classifications, it has only a descriptive role, leaving its clinical relevance open to debate. In addition, the actual incidence of iCSF-L in each anatomical area of the skull base during an endoscopic endonasal procedure is still unknown [11]. 

The aim of this manuscript is to provide a topographical map of iCSF-L of the skull base, comparing the incidence of each anatomical region with a group of post-traumatic and spontaneous CSF-L; in addition, the role of the skull base asymmetry is investigated as a factor for the occurrence of iCSF-L.

## 2. Materials and Methods

This is a monocentric retrospective observational study in which patients affected with iatrogenic, spontaneous, and post-traumatic CSF-L and surgically treated at our department between January 2001 and December 2023 were included. Exclusion criteria included primarily intradural procedures (e.g., tumor resection), CSF-L related to a tumor or post-radiotherapy, and congenital abnormalities of facial growth. It is of note that all iatrogenic cases were referred to our tertiary referral center to treat a CSF-L that occurred elsewhere.

Informed consent was obtained from each patient for treatment and use of de-identified clinical data for study purposes; the study, which was conducted according to the ethical standards of the Declaration of Helsinki revised in 2011, was approved by the French authorities (CNIL No. 2225234).

### 2.1. Patient Management and Study Variables

In all cases, diagnostic work-up included a pre-operative radiological study (high-speed spiral computed tomography [CT] using non-contrast axial 1.5 mm sections (high-resolution coronal and sagittal sections using specialized software, the Picture Archiving and Communication System [PACS]) and/or contrast-enhanced magnetic resonance imaging [MRI]), in which information was collected on (1) signs of intracranial hypertension; (2) bony modification of skull base structures; (3) localization of the skull base defect. The latter information was then compared to intra-operative findings and a combination of the two was used to clearly state the position of the dural defect.

For iCSF-Ls, data concerning the side, sex, age, Keros classification [7], Gera classification [5], distance of the anterior ethmoidal artery (AEA) from the skull base [9], frontal sinus (FS) pneumatization [8], and Thailand–Malaysia–Singapore score (TMS) [10] were analyzed. Specifically, measurements were conducted using the CareStream PACS (Carestream Health, Rochester, NY, USA) after having appropriately rectified the images. Each measurement was collected according to the respective original report [5,7,8,9,10]. In addition, the asymmetry between the two sides of the skull base was also collected and a specific class was added in each classification considered. Specifically, following previous reports [7,8,9], it was defined as >3 mm for the Keros classification and if a different class was present between the two sides considering the TMS, AEA distance, and FS pneumatization; conversely, considering the Gera classification, the cut-off to define the skull base asymmetry was arbitrarily set at >15°.

All data were compared to a uniform control group of patients consecutively treated for maxilla/fronto-ethmoidal inverted papilloma at our department with an endoscopic endonasal approach and who did not experience any surgical complications. The choice to select a uniform control group of inverted papillomas was taken to avoid any inclusion bias and, at the same time, consider a benign pathology that could have been complicated with iCSF-L.

Data were collected by two independent authors (I.D. and A.D.) and any discordance was resolved by a third analysis by the first author (A.V.).

Considering surgical management, the type of reconstruction (overlay vs. underlay), technique, number of layers, type of reconstruction, and application of mucosal flap/graft were collected. Data concerning pre-operative symptoms, side, use of post-operative antibiotics and acetazolamide (250 mg twice a day), post-operative lying, occurrence of pneumocephalus, and recurrence were also collected.

### 2.2. Study Objectives

The primary objective of this study was the creation of a topographic map of the skull base for CSF leak occurrence concerning the different etiology (iatrogenic, spontaneous, and post-traumatic) and the analysis of the difference between them; the secondary objective was the definition of anatomical factors that could predict the occurrence of iatrogenic CSF-L.

### 2.3. Statistical Analysis

Statistical analyses were performed using R v4.1.3. Data are reported as means ± standard deviations or percentages, as appropriate. Fisher’s exact test was used to evaluate the association of each CSF leak etiology with different qualitative clinical factors (radiological signs of intracranial hypertension, post-operative antibiotics, post-operative complications, recurrence, topographical localization of the CSF leak defect); in addition, it was used to compare the significance of each skull base classification between the case and control groups. The level of statistical significance was set at *p* < 0.05.

The receiver operating characteristic (ROC) curve was used to assess the prediction of an iatrogenic CSF leak. In order to compare the performance of the classifications, the area under the curve (AUC) was calculated and compared using Delong’s test [13]. AUC performance was considered null if AUC < 0.5, low if 0.5 ≤ AUC < 0.7, satisfactory if 0.7 ≤ AUC < 0.9, excellent if 0.9 ≤ AUC < 1, and perfect if AUC = 1.

## 3. Results

In total, 153 patients were included in this study, 37 iatrogenic, 103 spontaneous, and 13 traumatic CSF leaks. Descriptive characteristics of patients grouped by the type of CSF leaks are shown in Table 1. Among the iCSF-L patients, the original pathology for which they were originally operated was chronic rhinosinusitis with nasal polyps (12, 32.4%), chronic rhinosinusitis without nasal polyps (8, 21.6%), rhino/septoplasty (8, 21.6%), sinonasal mucocele (6, 16.3%), dacryocystorhinostomy, maxillary inverted papilloma, and nasal packing for an acute episode of epistaxis (1 case each, 2.7%, respectively).

Among all patients, pre-operative symptoms varied between watery rhinorrhea (87.6%), meningitis (7.2%), and headache (0.3%); considering pre-operative imaging, all spontaneous and post-traumatic cases underwent both MRI and CT. In the iatrogenic cases, 45.9% of cases underwent both and 54.1% were examined with a CT alone. When possible, the radiological signs of intracranial hypertension (e.g., empty sella, prominent subarachnoid space around the optic nerves, etc.) were analyzed and showed a significant difference between the three groups, with the spontaneous CSF leak group being the most affected (*p* < 0.001).

A detailed description of the reconstruction techniques used for the skull base defect goes beyond the purpose of this article, although in each case, the reconstruction was intra-operatively tailored based on the dimension and location of the dural defect, and no difference was noted between the techniques used in the three groups (*p* > 0.05), assuming, nevertheless, that in all cases a multi-layer technique was used, independently of the etiology of the CSF-L. Considering all cases, the intracranial intradural reconstruction (e.g., gasket seal, parachute technique, plug-in, etc.) was the most used (50.3%), followed by direct coagulation of the dural defect that was used as the “first layer” in 49.7% of cases. The reconstruction was reinforced in every case with a second layer of heterologous material (73.2%) or autologous material (26.8%, fascia lata, fibromucosa); as a third layer, in 66% of patients, a local mucosa flap and in 15%, a mucosal graft were applied, whereas in 15% of cases, no mucosal layer was applied. The overall success rate of reconstruction did not show differences between the type of CSF leak (Table 1, *p* > 0.05).

No bed lying was prescribed in any case, and acetazolamide (250 mg twice a day) was given only in cases of intracranial hypertension. In all patients, intra-operative antibiotic prophylaxis was prescribed, whereas it was rarely continued post-operatively (six cases in total), only if infection of the sinonasal mucosa was present.

Post-operative complications (meningitis, cerebral abscess, intra-dural hematoma, and post-operative infection) were significantly more prevalent in post-traumatic cases (*p* < 0.01).

### 3.1. Analysis of Topographic Features

**Figure 1:**
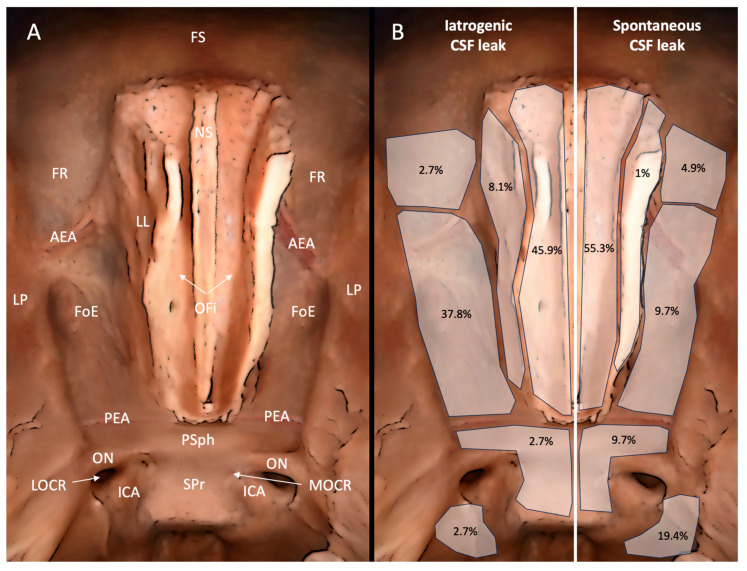
Graphical representation of the skull base (**A**) in which the incidence of iatrogenic (**left** side) and spontaneous (**right** side) CSF leaks is shown in each anatomical region considered (**B**). OFi = olfactory fissure, FoE = fovea ethmoidalis, AEA = anterior ethmoidal artery, PEA = posterior ethmoidal artery, LP = lamina papyracea, FS = frontal sinus, FR = frontal recess, PSph = planum sphenoidalis, ON = optic nerve, ICA, internal carotid artery, LOCR = lateral optic–carotid recess, MOCR = medial optic–carotid recess, LL = lateral lamella, NS = nasal septum, SPr = sellar prominence.

A description of the skull base regions involved according to the nature of the CSF leak is displayed in contingency Table 2. When compared, a significantly different association was noted (*p* < 0.001) between the nature of the CSF leak and involved areas, both in general and in two-by-two analyses, reinforcing the different topographical incidence of the dural defect in each type of CSF leak analyzed (Figure 1).

### 3.2. Iatrogenic CSF Leak and Skull Base Anatomy

**Figure 2:**
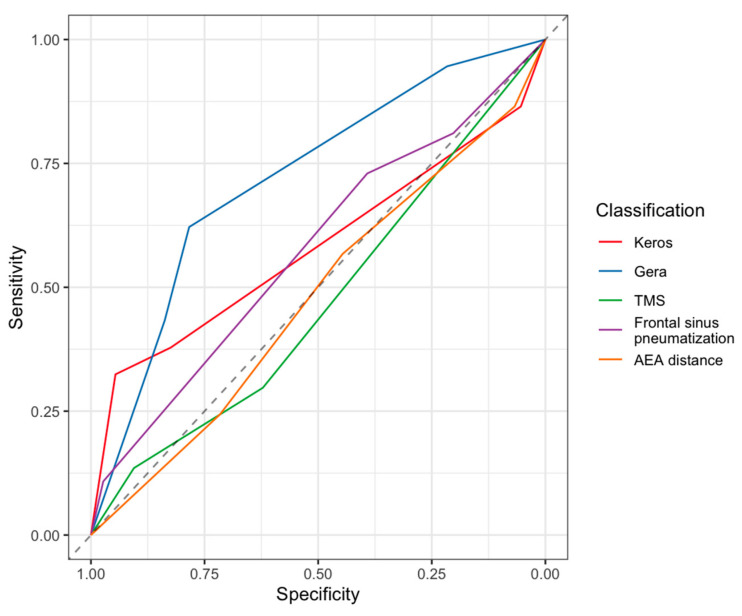
Receiver operating characteristic (ROC) curve to assess the prediction of iatrogenic CSF leaks taking into consideration the Keros, Gera, distance of the anterior ethmoidal artery (AEA) from the skull base, frontal sinus pneumatization, and Thailand–Malaysia–Singapore score classification (TMS).

**Figure 3:**
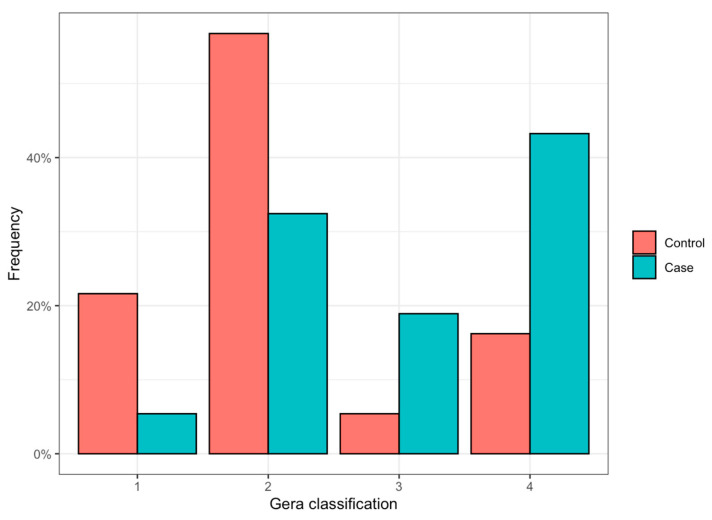
Bar chart representing the distribution of the Gera classification among case and control groups.

When considering the 37 iCSF-L cases and comparing them with the 74 control patients, no significant difference was noted between sex (M/F, 24:13 in iCSF-L group vs. 43:31 in control group, *p* > 0.05), side (right/left, 17:20 in iCSF-L group vs. 34:40 in control group, *p* > 0.05), and age (47.3 years, SD 15.2 years, in in iCSF-L group vs. 58.4 years, SD 12.1 years, in control group, *p* > 0.05). Conversely, among all classifications considered to assess the risk of skull base injury, the Keros and Gera classifications were significantly different between the two groups (*p* = 0.02 and <0.001, respectively). When the AUC (95% CI) was considered, the Gera, Keros, distance of the anterior ethmoidal artery from the skull base, frontal sinus pneumatization, and Thailand–Malaysia–Singapore score (TMS) classifications were, respectively, 0.719 (0.624–0.813), 0.581 (0.469–0.692), 0.478 (0.367–0.589), 0.574 (0.471–0.677), and 0.471 (0.373–0.569) (Table 3 and Figure 2). When compared using DeLong’s test, a significant difference was noted with the Gera classification (Figure 3), which was the most reliable compared to TMS (*p* < 0.001), Keros classification (*p* = 0.048), and AEA distance (*p* = 0.018). Conversely, the difference between the Gera and frontal sinus pneumatization classification was not significant (*p* = 0.055).

## 4. Discussion

The main finding of our study is that dural transgressions during sinonasal procedures occurred more frequently at the level of the cribriform plate (45.9%) and ethmoidal roof (37.8%), and only 8.1% of cases were localized at the level of the lateral lamella. In addition, after having assessed the asymmetry of the skull base, among all the available classifications that grade the risk of iCSF-L, the Gera classification was superior (AUC = 0.719, *p* < 0.05), highlighting that asymmetry plays a major role compared to the control group (43.3% vs. 16.2%). To the authors’ knowledge, this study is the first of its kind since we have not only provided a topographical map of iCSF-L occurrence but also explored the clinical role of skull base asymmetry by comparing the available classifications.

iCSF-L is a rare but challenging complication that is known to occur in more thin and delicate regions such as the lateral lamella and the posterior ethmoidal roof [14]. Indeed, the first is known to be the riskiest point for dural transgression during endonasal procedures, particularly when the middle turbinate is manipulated; the second is said to be at risk due to its peculiar inferior slope orientation to the sphenoidal sinus [2,15]. Nevertheless, our results show that iCSF-Ls occur at the level of the lateral lamella in only 8.1% of cases, whereas the majority of dural transgressions were located at the level of the cribriform plate (45.9%) and antero-posterior ethmoidal roof (37.8%) (Figure 1). The central role of the latter two regions in iCSF-L has been already described by Tang et al., reinforcing our results [11]. Indeed, the dural transgression in iatrogenic cases occurs in different regions compared to spontaneous and traumatic cases (*p* < 0.001).

However, if the motivation of iCSF-L occurrence at the level of the ethmoidal roof is intuitive and principally due to its variable thickness and slope, both at its anterior and posterior portion, the involvement of the cribriform plate during endonasal procedures is less understandable. Indeed, the olfactory fossa is more frequently known to be the principal region for spontaneous CSF, which is confirmed by our results that show its involvement in 55.3% of spontaneous cases, due to its extreme fragility and presence of dural evagination [16]. However, the cribriform plate stays outside the ethmoidal corridor and, consequently, its region should not be approached not only during rhino/septoplasty but also during ethmoidectomies, so that it is more likely that dural transgression of the cribriform plate may be justified by surgical disorientation or technical error, facilitated by extensive middle turbinate resection [17]. This theory is reinforced by the original pathology of patients with iCSF-L, which could have been considered “at risk” in twenty cases (twenty chronic rhinosinusitis, 54%) but not for the remaining seventeen (48%, eight rhino/septoplasty, six mucocele, one dacryocystorhinostomy, one maxillary inverted papilloma, and one nasal packing). This evidence underlines that extensive anatomical knowledge and increased surgical experience may reduce the incidence of iCSF-L [11]. 

Finally, it is of note that the lateral wall of the sphenoidal sinus is not of importance in iCSF-L (2.7%), but it plays a major role in cases with a spontaneous etiology (19.7%), which can be justified by the presence of Sternberg’s canal that could facilitate a CSF in the case of intracranial hypertension [18]. 

Nonetheless, independently of the surgical experience, iCSF-L represents a possible complication in endonasal procedures that might be related to anatomical variations in the anterior skull base [6]. In the past two decades, several classifications have described potential risk factors for dural transgression, namely the Keros, Gera, TMS, and AEA distance classifications and frontal sinus pneumatization [5,7,8,9,10]. The Keros (1962) classification was the first to be introduced and distinguishes three types of cribriform fossa depending on its depth: the idea behind it was that the more pronounced the depth of the cribriform cleft, the thinner the lateral lamella, thus exposing this area to potentially more frequent iatrogenic injuries [7]. Even though widely used, several authors have highlighted the limitations of this classification in fully describing the shape of the skull base and predicting the risk of iCSF-L [19,20]. The TMS and Gera classifications were described to resolve the abovementioned problem by classifying, respectively, the relation between the height of the ethmoidal roof and the depth of the cribriform plate and the angulation of the lateral lamella to the cribriform plate. In particular, the basis of the Gera classification was that a more pronounced slope of the anterior skull base may predispose patients to dural transgression at the level of the medial portion of the skull base when dissecting the antero-medial ethmoidal cells, with class III (<45°) being the riskiest [5,21,22,23]. Finally, frontal sinus pneumatization and AEA distance classification may affect the anatomy of the anterior skull base and length of the lateral lamella, predisposing patients to surgical complications [8]. In addition, some authors have added a potential radiological factor for iCSF-L: asymmetry of the skull base between the two sides. Indeed, the presence of this asymmetry could lead the surgeon to incorrect orientation and facilitate complications such as dural injury. In pre-clinical settings, skull base asymmetry has been found in 8.6–93% of patients [12,21,22,23,24]. However, its clinical applicability has never been defined and, additionally, the definition of asymmetry is rarely uniform (i.e., difference of Keros depth > 2/3 mm), leaving its interpretation open to debate.

In our series, the prevalence of skull base asymmetry among each classification varied between 13.4 and 43.3% and between 5.4 and 20.3%, respectively, in the case and control groups (Table 3). Nevertheless, when considering all cases with logistic regression analysis, only the Keros and Gera classifications, integrated with the notion of skull base asymmetry, showed a significant difference between the iCSF-L and control groups (*p* < 0.001). Moreover, when the prediction of iCSF-L (ROC curve, Figure 2) was assessed, the Gera classification was the most reliable (AUC = 0.719, Table 3, Figure 3) and significantly different from the Keros, AEA distance, and TMS classification (*p* < 0.05). No significant difference was noted with the frontal sinus pneumatization classification (*p* = 0.055), even if a trend in favor of the Gera classification was noted (AUC = 0.719 and 0.574, respectively). These results, together with the notion of skull base asymmetry (Figure 3), underline that the slope of the lateral lamella (Gera classification) is the most reliable factor in predicting the risk of dural transgression.

This study has some limitations: first of all, its retrospective nature, and second, the absence of a reliable matched control group that would have strengthened the role of skull base asymmetry in multivariate analysis. Finally, considering that all cases were referred to our center to manage iCSF-L, the actual incidence of this complication cannot be determined.

## 5. Conclusions

Our results demonstrate that the regions involved in iCSF-L are different compared to spontaneous and traumatic cases, with the cribriform plate (45.9%) and ethmoidal roof (37.8%) being the most involved. Additionally, after having assessed the asymmetry of the skull base, among all classifications that may predict the risk of iatrogenic dural transgression, the Gera classification was the most reliable.

## Figures and Tables

**Table 1:** Characteristics of patients by the type of cerebrospinal fluid (CSF) leak. Bold characteristics were significantly different in statistical analysis: intracranial hypertension, *p* < 0.001; post-operative complications, *p* = 0.005.

	Iatrogenic CSF Leak	Spontaneous CSF Leak	Traumatic CSF Leak
No. patients	37	103	13
Age at surgery. Mean (SD)	47.5 (15.2)	51.9 (15.1)	50.9 (15.2)
Sex, male/female	24:13	31:72	10:3
Side, right/left	17:20	47:56	7:6
**Radiological sign of intracranial hypertension, N (%)**	**2 (5.4%)**	**60 (58.3%)**	**3 (23.1%)**
Post-operative antibiotics, N (%)	2 (5.4%)	3 (2.9%)	1 (7.7%)
**Post-operative complications, N (%)**	**1 (2.7%)**	**2 (1.9%)**	**3 (23.1%)**
Recurrence, N (%)	1 (2.7%)	4 (3.9%)	1 (7.7%)

**Table 2:** Topographical description of the skull base defect causing the cerebrospinal fluid (CSF) leak, expressed as numbers and percentages.

	Iatrogenic CSF Leak	Spontaneous CSF Leak	Traumatic CSF Leak
Posterior plate of the frontal sinus/frontal recess	1 (2.7%)	5 (4.9%)	2 (15.4%)
Fovea ethmoidalis	14 (37.8%)	10 (9.7%)	4 (30.8%)
Lateral lamella	3 (8.1%)	1 (1%)	1 (7.7%)
Cribriform plate	17 (45.9%)	57 (55.3%)	1 (7.7%)
Planum sphenoidalis and posterior wall of the sphenoidal sinus	1 (2.7%)	10 (9.7%)	3 (23.1%)
Lateral wall of the sphenoidal sinus	1 (2.7%)	20 (19.4%)	2 (15.4%)

**Table 3:** Analysis of skull base predisposing factors in the iatrogenic CSF leak cohort compared to the control group. FS = frontal sinus, AEA = anterior ethmoidal artery, SB = skull base, AUC = area under the curve, OF-CP = distance from the orbital floor to the cribriform plate; OF-ER = distance from the orbital floor to the ethmoidal roof.

	Iatrogenic CSF Leak Group N = 37	Control Group N = 74	AUC (95% CI)
**Gera classification**			
Class I (>80°, low risk)	2 (5.4%)	16 (21.6%)	0.719 (0.624–0.813)
Class II (45–80°, medium risk)	12 (32.4%)	42 (56.8%)	
Class III (<45°, high risk)	7 (18.9%)	4 (5.4%)	
Class IV (asymmetry > 15°)	16 (43.3%)	12 (16.2%)	
**Keros classification**			
Type I (1–3 mm)	5 (13.5%)	4 (5.4%)	0.581 (0.469–0.692)
Type II (4–7 mm)	18 (48.7%)	57 (77%)	
Type III (8–16 mm)	2 (5.4%)	9 (12.2%)	
Type IV (asymmetry)	12 (32.4%)	4 (5.4%)	
**Frontal sinus pneumatization**			
Aplasia–hypoplasia	4 (10.8%)	2 (2.7%)	0.574 (0.471–0.677)
Pneumatization medial to the midorbital line	23 (62.2%)	43 (58.1%)	
Hyperplasia	3 (8.1%)	14 (18.9%)	
Asymmetry	7 (18.9%)	15 (20.3%)	
**AEA distance from the SB**			
AEA included in the SB	9 (24.4%)	21 (28.4%)	0.478 (0.367–0.589)
AEA under the SB	12 (32.4%)	20 (27%)	
AEA runs freely at a distance below the SB	11 (29.8%)	28 (37.8%)	
Asymmetry	5 (13.4%)	5 (6.8%)	
**Thailand–Malaysia–Singapore score (TMS)**			
Type 1 (OF-CP and OF-ER >10 mm)	26 (70.3%)	46 (62.1%)	0.471 (0.373–0.569)
Type 2 (OF-CP or OF-ER < 10 mm)	6 (16.3%)	21 (28.4%)	
Type 3 (OF-CP and OF-ER < 10 mm)	0 (0%)	0 (0%)	
Type 4 (asymmetry)	5 (13.4%)	7 (9.5%)	

## Data Availability

The data presented in this study are available on request from the corresponding author.
